# Evaluation of Sample Stability and Automated DNA Extraction for Fetal Sex Determination Using Cell-Free Fetal DNA in Maternal Plasma

**DOI:** 10.1155/2013/195363

**Published:** 2013-10-07

**Authors:** Elena Ordoñez, Laura Rueda, M. Paz Cañadas, Carme Fuster, Vincenzo Cirigliano

**Affiliations:** ^1^Departament de Genètica Molecular, Labco Diagnostics, c/Londres 28, 08029 Barcelona, Spain; ^2^Unitat de Biologia, Departament de Biologia Cellular, Fisiologia i Immunologia, Universitat Autònoma de Barcelona, 08193 Barcelona, Spain

## Abstract

*Objective*. The detection of paternally inherited sequences in maternal plasma, such as the SRY gene for fetal sexing or RHD for fetal blood group genotyping, is becoming part of daily routine in diagnostic laboratories. Due to the low percentage of fetal DNA, it is crucial to ensure sample stability and the efficiency of DNA extraction. We evaluated blood stability at 4°C for at least 24 hours and automated DNA extraction, for fetal sex determination in maternal plasma. *Methods*. A total of 158 blood samples were collected, using EDTA-K tubes, from women in their 1st trimester of pregnancy. Samples were kept at 4°C for at least 24 hours before processing. An automated DNA extraction was evaluated, and its efficiency was compared with a standard manual procedure. The SRY marker was used to quantify cfDNA by real-time PCR. *Results*. Although lower cfDNA amounts were obtained by automated DNA extraction (mean 107,35 GE/mL versus 259,43 GE/mL), the SRY sequence was successfully detected in all 108 samples from pregnancies with male fetuses. *Conclusion*. We successfully evaluated the suitability of standard blood tubes for the collection of maternal blood and assessed samples to be suitable for analysis at least 24 hours later. This would allow shipping to a central reference laboratory almost from anywhere in Europe.

## 1. Introduction

The use of cell-free fetal DNA (cfDNA) in maternal plasma is part of the daily routine in several genetic centers for noninvasive fetal sex determination, RhD genotyping, and more recently aneuploidy screening [1–5]. Although cfDNA represents a very low percentage of the total amount of free DNA circulating in plasma (3–6%), paternally inherited sequences absent in the mother can easily be detected in plasma samples from pregnant women from the first trimester of pregnancy. Non-invasive fetal sex determination has become a very useful tool in the management of pregnancies at risk of X-linked inherited disorders, as it allows reducing the need of invasive procedures [6–9]. 

However, the low proportion of ffDNA present in maternal plasma still poses some technical difficulties related to sample stability during transport and DNA extraction methods. Different effects of blood processing protocols on the amount of retrievable cfDNA have been reported, and the time between venopuncture and cfDNA recovery has also been shown to favor blood cells haemolysis, thus, reducing the fetal DNA fraction [10–12].

Different strategies have been suggested to try stabilizing blood samples to improve cfDNA extraction, mainly limiting the time before plasma separation or eventually using formaldehyde [13–17]. Also, specific collection tubes stabilizing blood samples are now commercially available, although being not approved yet for diagnostic purposes [18].

We evaluated the suitability of blood collection in standard CE marked EDTA-K tubes and their storage at 4°C for at least 24 hours, to detect fetal SRY sequences in maternal plasma. 

This could allow easy and cost-effective sample collection at local clinics, providing enough time for samples shipping to a central reference lab to be processed on the following day. 

We also evaluated the suitability of using an automated DNA extraction method for low cost and high throughput analysis by comparing cfDNA yield obtained with a reference manual DNA extraction procedure.

## 2. Materials and Methods

Blood samples were collected in 2 × 3 mL EDTA-K tubes from over 400 pregnant women between 11th and 13th weeks of gestation (mean: 12) just before undergoing an invasive procedure (CVS) for prenatal diagnosis of chromosome abnormalities. Blood samples were kept at 4°C before being sent to the laboratory; same storage conditions were also kept upon reception for at least 24 hours from venopuncture. Plasmas were separated from the cellular fraction by a first centrifugation at low speed (10 minutes at 1200 g) and second high speed centrifugation for 10 minutes at 16000 g and 4°C. Supernatants were stored at −20°C until DNA extraction. All CVS samples were analysed by QF-PCR for rapid prenatal diagnosis of chromosomes X, Y, 21, 18, and 13 aneuploidies with results made available to referring physicians within 24 hours from sampling [19].

Based on QF-PCR results, a total of 108 plasmas were selected from women carrying normal male fetuses so that DNA extraction efficiency could be monitored by real-time PCR quantification of the SRY gene. Fifty more samples from women carrying normal female fetuses were also selected as negative controls.

The first batch of 50 samples from male fetuses was processed in duplicate using manual and automated extractions. DNA extractions were performed from 500 *µ*L of plasma using the QIAamp DSP virus kit (QIAGEN Inc.) slightly modifying the manufacturer protocol, with a final DNA elution of 55 *µ*L. A total of 850 *µ*L of plasma from the same sample was also extracted using the COBAS AmpliPrep-total nucleic acid isolation (TNAI) kit on the COBAS AmpliPrep DNA/RNA extractor (Roche Diagnostics) using a final elution volume of 75 *µ*L. 

All remaining samples were processed with the automated method; DNAs were kept at −20°C until PCR analysis.

Extracted DNAs were tested in duplicates, and cfDNA amounts were evaluated by absolute quantification of the SRY gene using real-time PCR as previously described (Zhong et al., 2001) [20]. Positive SRY amplification with a threshold cycle value (Ct) <42 was expected in at least one of the duplicates from male pregnancies, while undetectable amplification was expected in female pregnancies used as negative controls. 

Standard quantification curves were generated using human male DNA at 10 ng/*µ*L, 1 ng/*µ*L, 0,1 ng/*µ*L, and 0,01 ng/*µ*L concentrations, and cfDNA yield (Genomic Equivalents/mL of plasma) was calculated for all samples, and results, were compared for manual and automated DNA extraction.

## 3. Results

DNA extraction following whole blood storage at 4°C for at least 24 hours was successful using both manual and automated procedures, and SRY amplification was detected in all 108 plasma samples from pregnant women with male fetuses. No false negative results were observed; SRY amplification was positive in both duplicates from all manual extractions and in 102/108 cases from automated procedure. 

**Figure 1:**
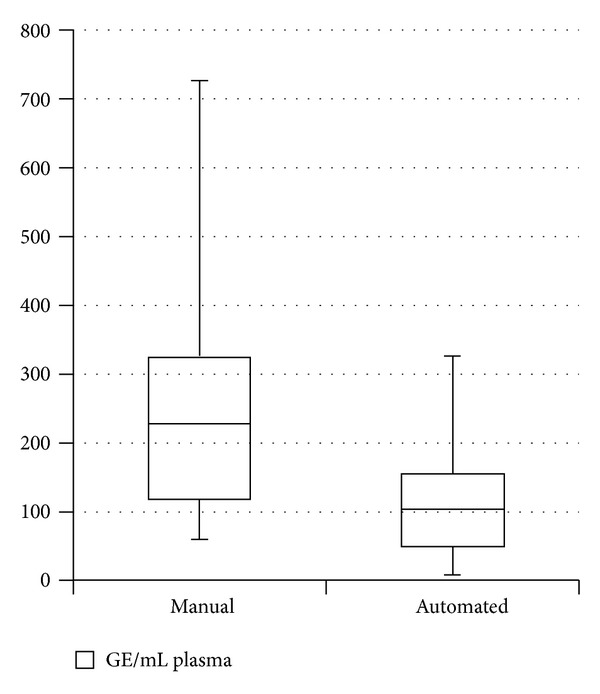
Genomic equivalents of cfDNA in 50 samples from male fetuses extracted with COBAS Ampliprep and QIAGEN Viral DSP procedures.

As shown in Table 1, manual DNA extraction resulted in lower Ct values for the SRY amplification (mean Ct = 36,59) than automated procedure (mean Ct = 37,74). Overall, cfDNA amounts were higher using manual extraction compared with the automated system, with a mean quantity of cfDNA of 259.43 GE/mL of plasma (range: 61,05–725.33) and 107,35 GE/mL (range: 10,28–327,06), respectively (Figure 1).

Only 6 samples failed one of the duplicates, all extracted with the automated procedures, and in 4/6 cases, the cfDNA amount was slightly lower than the observed mean.

As shown in Figure 1, more variability in cfDNA amounts was observed for samples obtained by manual DNA extraction compared with the automated procedure.

SRY detection and quantification were also possible in the remaining 58 samples, and no SRY amplification was observed for the 50 DNAs derived from women carrying female fetuses, all tested only with the automated procedure.

## 4. Discussion

Cell-free fetal DNA is present in low proportion in maternal plasma; thus, an efficient DNA extraction method and a robust PCR assay are crucial to detect paternally inherited sequences such as the SRY or RhD genes. Apart from different biological factors such as maternal weight or placental pathology [5, 21–24] the fetal fraction can also decrease as a result of maternal blood cells lysis in the time between blood draw and plasma separation prior to DNA extraction [14, 16, 25–27]. This might be a limitation for a central reference diagnostic laboratory offering cfDNA analysis for fetal sexing or RhD genotyping, as samples must reach the laboratory within the shortest possible period of time. 

We evaluated the cfDNA stability when using standard EDTA-K tubes to collect maternal blood, by only separating maternal plasma from the cell pellets after enough time to eventually allow samples to be shipped from almost anywhere in Europe to a reference diagnostic laboratory. 

 The SRY sequence was detected in all plasmas from male pregnancies, despite samples being kept at 4°C for at least 24 hours from sampling. 

The QIAamp DSP Virus Kit (QIAGEN, Hilden, German) has been shown as one of the most efficient DNA extraction methods for cfDNA analysis [14, 28, 29]; however, the use of an automated extractor, would ideally help in reducing risks of sample mishandling and cross contamination, while also contributing to a reduction of costs [30–35]. 

We successfully evaluated a protocol for the COBAS AmpliPrep automated system to be used for cfDNA extraction from maternal plasma samples collected in the 1st trimester of pregnancy. This is a CE/FDA approved system routinely used for the detection and quantification of viral nucleic acids in clinical samples with proven efficiency to allow PCR detection of targets with concentrations as low as 5 copies per mL. Despite the lower cfDNA yield observed in comparison with the QIAGEN DSP Virus Kit, the automated DNA extraction provided lower variability in concentrations range and proved to be robust enough to not affect the rtPCR efficiency allowing detecting SRY for at least one of the replicates in all male cases. 

Automated DNA extraction allows high throughput of samples (up to 72 samples/run) in a closed system greatly reducing the risk of cross contamination, and it also requires little manipulation thus reducing overall costs and hands on time. 

Specific cfDNA collection tubes have recently been developed in order to stabilise the sample at room temperature by limiting maternal blood cells lysis during transport. This solution is now routinely used for cfDNA screening of fetal aneuploidies in the US [18]. However, these tubes are more expensive than most rtPCR, while this would not be an issue for next generation sequencing based tests, it represents more than doubling overall laboratory costs for fetal sexing and RhD genotyping. Furthermore, cfDNA collection tubes are still labelled *for research use only*, thus, not suitable to collect diagnostic samples in European countries, unless being previously fully validated by each laboratory and for each different intended use. 

## 5. Conclusion

We successfully evaluated the suitability of using standard CE marked blood tubes for the collection of maternal blood and the stability of samples following storage for at least 24 hours. This was coupled with an automated DNA extraction, to further simplify the procedure with a view to cost reduction and to simplify its introduction in the daily routine of clinical diagnostic laboratories. This approach was shown to be robust enough to obtain cfDNA from samples collected in the 1st trimester of pregnancy allowing fetal sex to be correctly identified in all cases analysed in the course of this study. 

## Figures and Tables

**Table 1:** Efficiencies of manual and automated cfDNA extractions. Despite consistently producing higher Ct values (lower cfDNA amounts), automated DNA extraction of samples collected in the 1st trimester of pregnancies produced cfDNA amounts well within the detection limits of rtPCR.

	Manual DNA extraction (QIAGEN DSP) *N* = 50	Automated DNA extraction (COBAS AmpliPrep) *N* = 108
Ct values	34,31–44,87 (36,59)	34,17–46 (37,74)
cfDNA amounts (GE/mL)	259,43 (61,05–725,33)	107,35 (10,28–327,06)
